# Supplementary figures and images for: New distributional records of the Samana least gecko (Sphaerodactylus samanensis, Cochran, 1932) with comments on its morphological variation and conservation status
Source: PeerJ. 2021 Jan 11;9:e10404. doi: 10.7717/peerj.10404 (PMC7808264; doi:10.7717/peerj.10404)

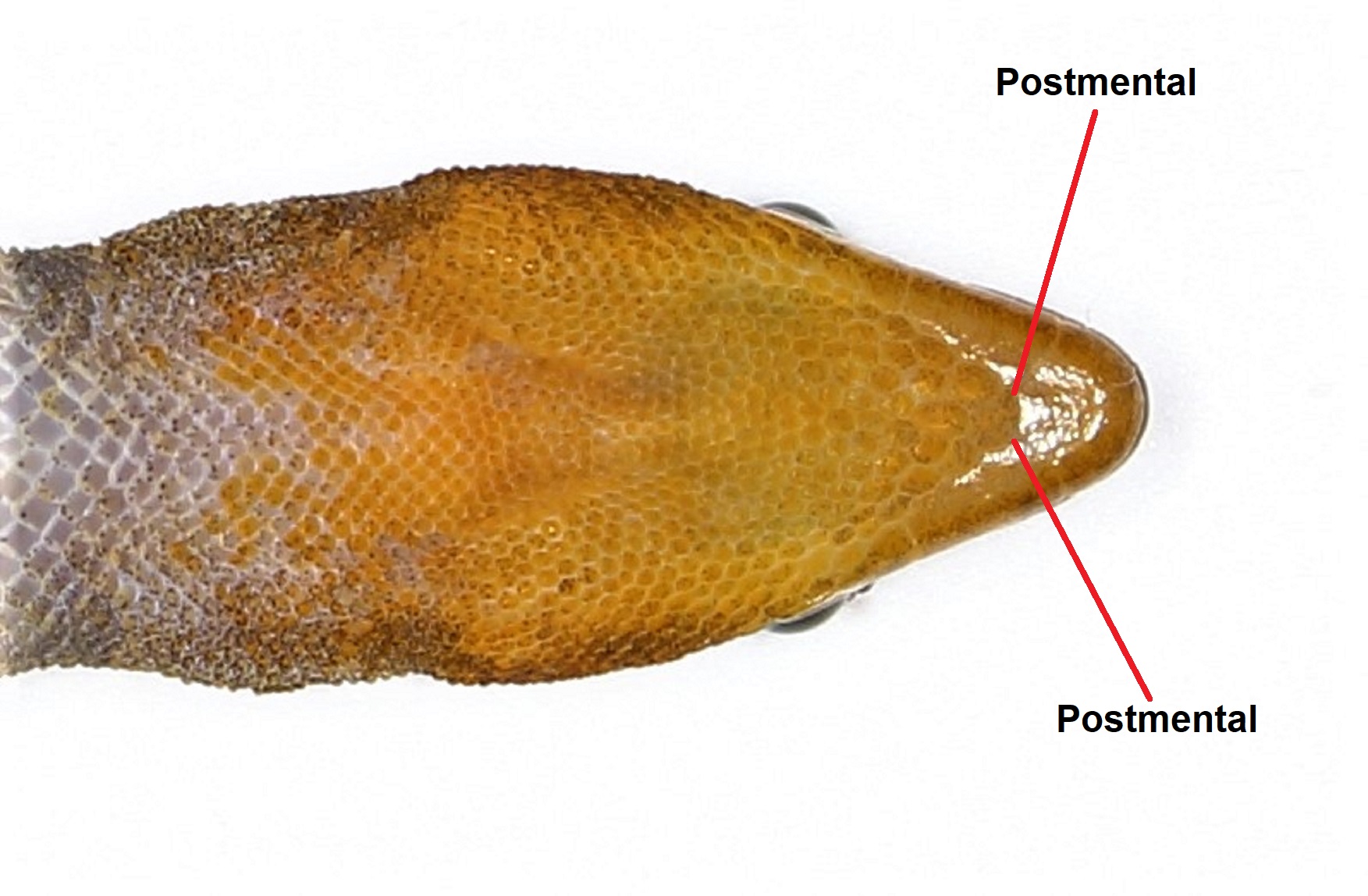

Supplement: Supplemental Information 2 [file peerj-09-10404-s002.jpg]

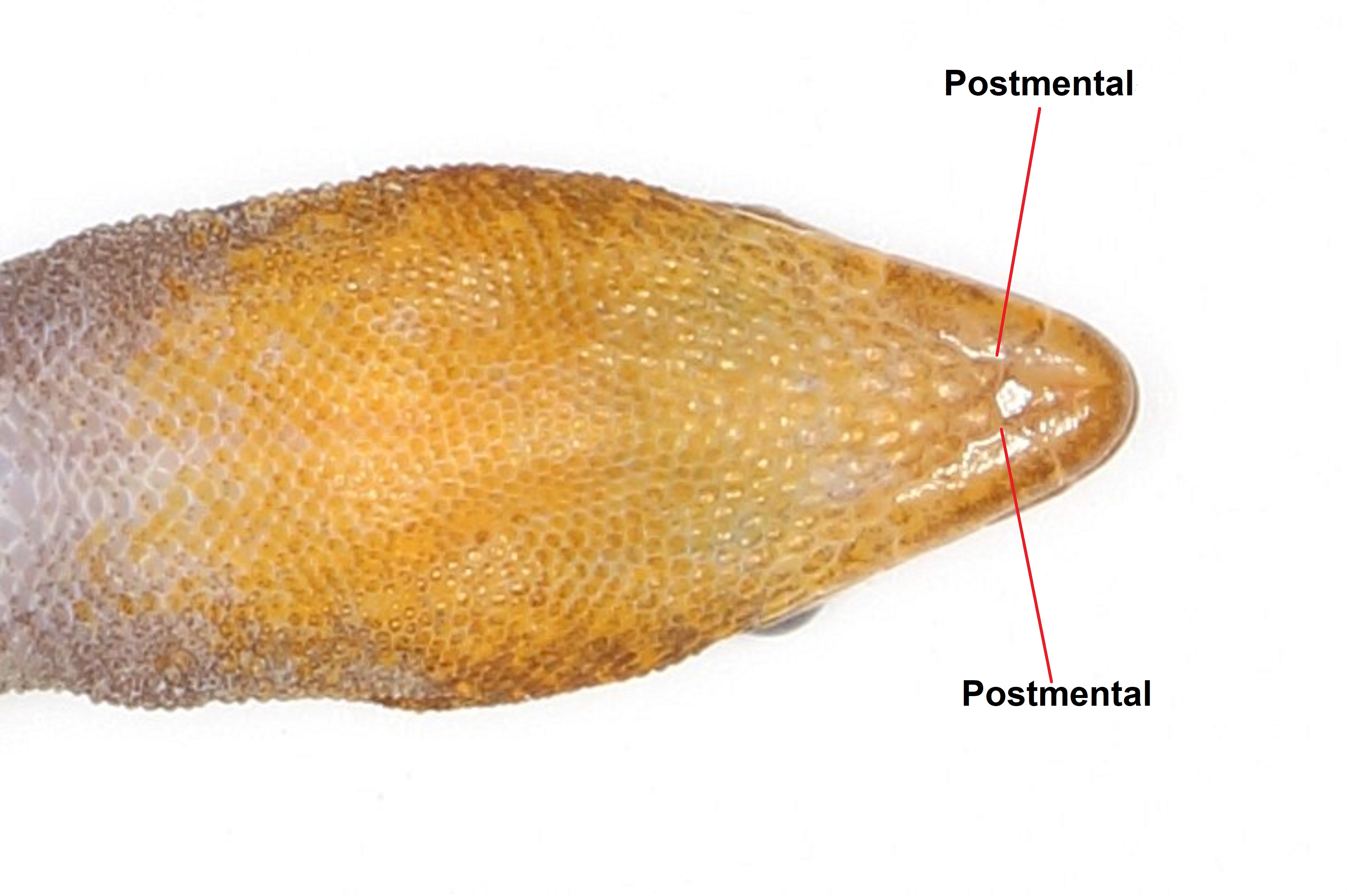

Supplement: Supplemental Information 3 [file peerj-09-10404-s003.jpg]

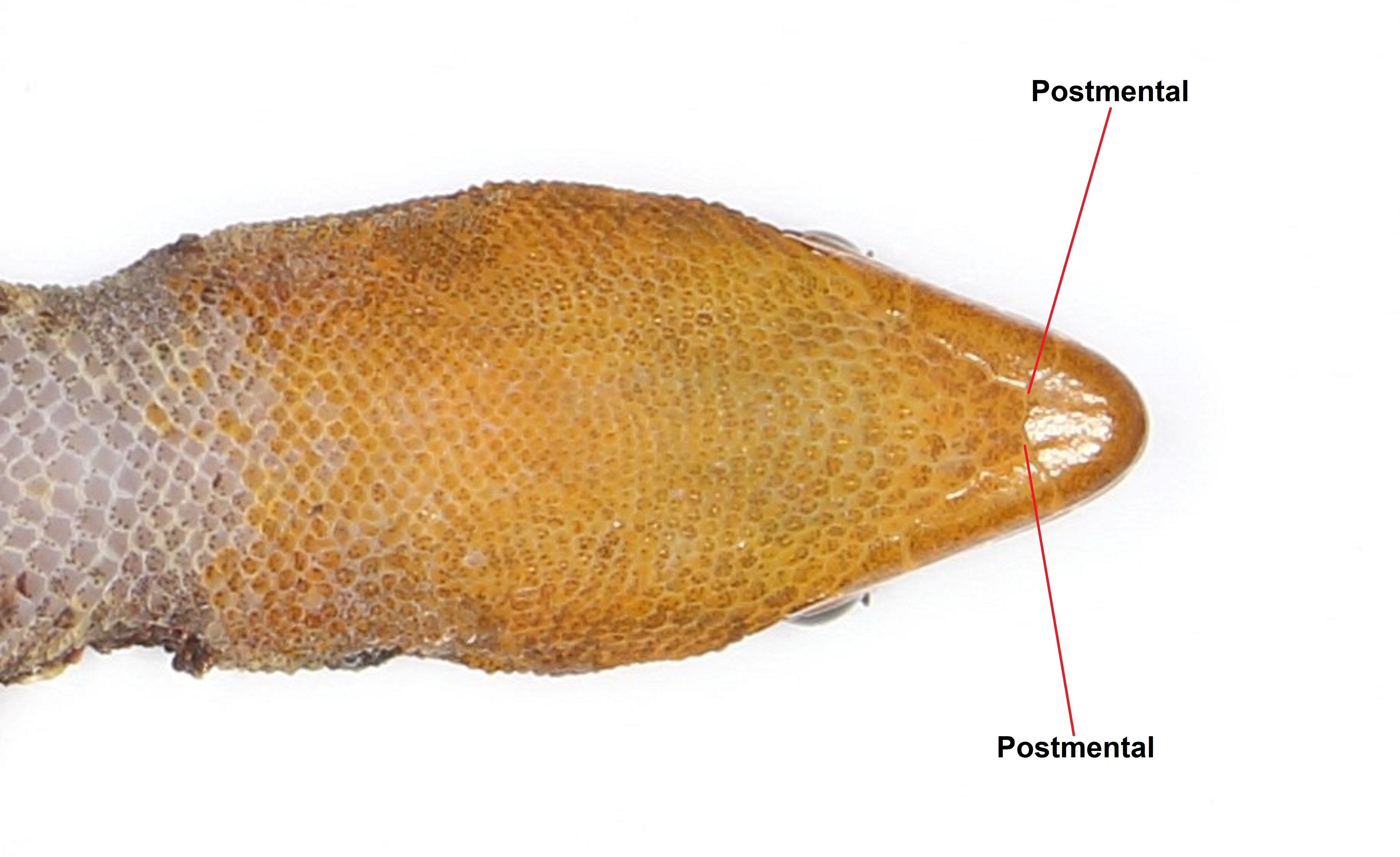

Supplement: Supplemental Information 4 [file peerj-09-10404-s004.jpg]

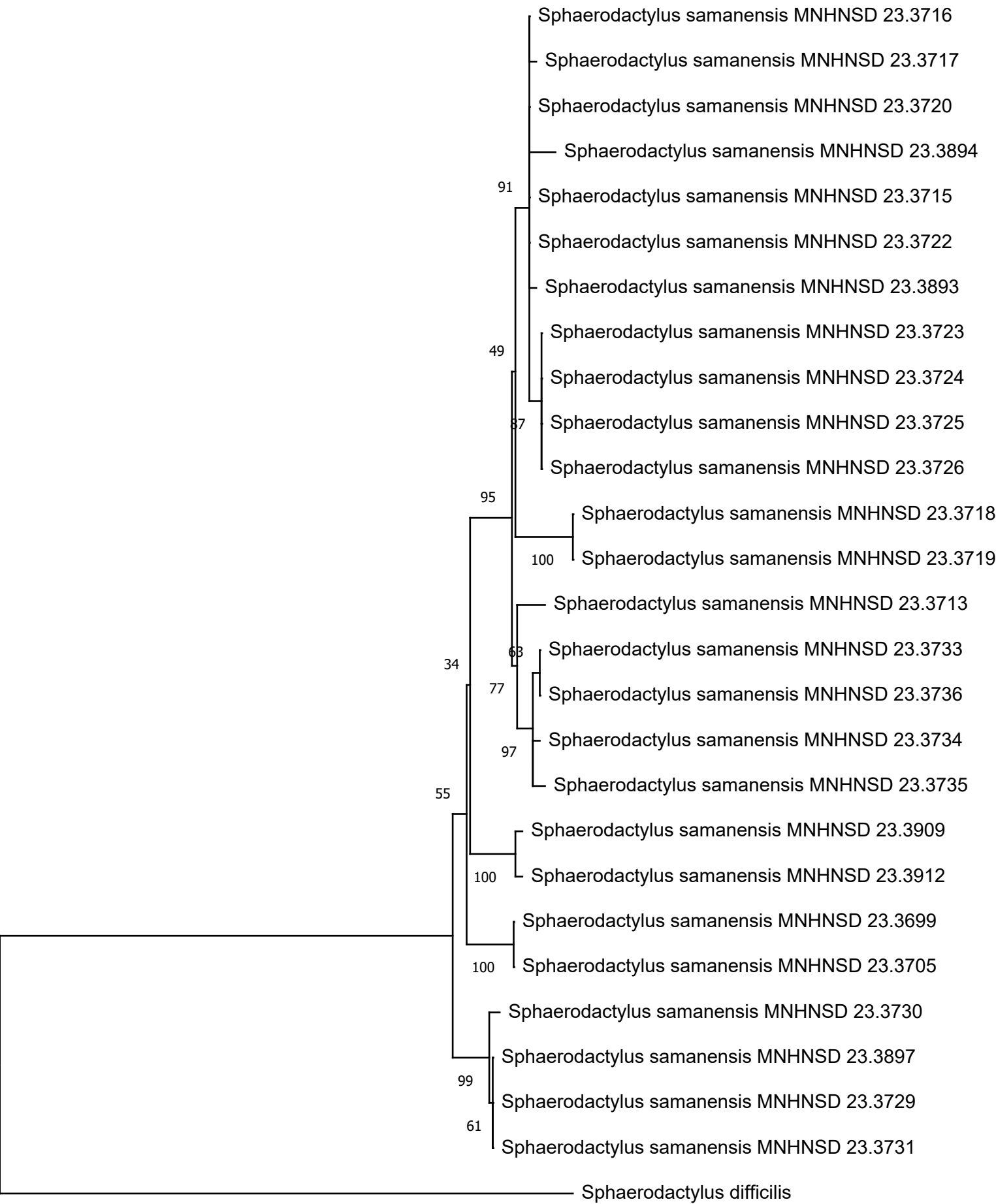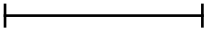

0.020

Supplement: Supplemental Information 6 [file peerj-09-10404-s006.pdf]
