# Supplementary material for: New distributional records of the Samana least gecko (Sphaerodactylus samanensis, Cochran, 1932) with comments on its morphological variation and conservation status
Source: PeerJ. 2021 Jan 11;9:e10404. doi: 10.7717/peerj.10404 (PMC7808264; doi:10.7717/peerj.10404)
Supplement: Supplemental Information 5 [file peerj-09-10404-s005.docx]

| ***Sphaerodactylus samanensis*'s specimens stored in the Museo Nacional de Historia Natural “Prof. Eugenio de Jesús Marcano” Santo Domingo, Dominican Republic** | | | | | |
| --- | --- | --- | --- | --- | --- |
| Locality | Province | Coordinates (lat, lon) | Altitud (m) | Specimens voucher | |
|  |  |  |  | Males | Females |
| Caño Hondo (Los Haitises National Park) | Hato Mayor | 19.05894, -69.4633 | 44 | MNHNSD 23.3715−16, 23.3718 | MHNHSD 23.3717, 23.3719−20, 23.3722, 23.3893 |
| Cueva Casa Grande | Monte Plata | 19.04214, -69.72787 | 225 | MNHNSD 23.3723 | MNHNSD 23.3724−26, 23.3894 |
| Batey Piedra | Sanchez Ramirez | 19.06997, -69.90815 | 35 | MNHNSD 23.3895−96, 23.3899 | MNHNSD 23.3729−31, 23.3897−98, 23.3900−02 |
| Chacuey Bajo | Sanchez Ramirez | 19.10689, -70.04149 | 115 | MNHNSD 23.3733−35, 23.3905 | MNHNSD 23.3736, 23.3903−04, 23.3906−08 |
| Pueblo Viejo Mine | Sanchez Ramirez | 18.92348, -70.15423 | 195 | MNHNSD 23.3699, 23.3706−07, 23.3909 | MNHNSD 23.3697−98, 23.3701−05, 23.3910−14 |
| Cueva de Sanabe (Aniana Vargas National Park) | Sanchez Ramirez | 19.00004, -70.23809 | 257 | MNHNSD 23.3713 | MNHNSD 23.3712 |
